# Building a resistance to ignition testing device for sunglasses and analysing data: a continuing study for sunglasses standards

**DOI:** 10.1186/s12938-017-0404-1

**Published:** 2017-09-21

**Authors:** Renan Magri, Mauro Masili, Fernanda Oliveira Duarte, Liliane Ventura

**Affiliations:** Electrical Engineering Department, Engineering School of São Carlos, University of São Paulo, Av. Trabalhador Sãocarlense 400, São Carlos, SP 13566-590 Brazil

**Keywords:** Sunglasses standards, ISO 12312-1, Resistance to ignition test, Sunglasses frames and lenses

## Abstract

**Background:**

Sunglasses popularity skyrocketed since its advent. The ongoing trend led to the creation of standards to protect consumers from injuries and secondary hazards due to spectacles use. In Brazil, the corresponding standard is NBR ISO 12312-1:2015 and since there is no mandatory testing, evaluating sunglasses performance provides an insight into compliance with the standard. In a continuing revision of sunglasses standards requirements, resistance to ignition is one of the concerns, since sunglasses should be protected from burning into flames at a pre-determined temperature, which may protect user of getting their sunglasses into flames if some, cigarette sparks reaches the spectacles, as an example. This paper describes the building of a resistance to ignition system and the results of 410 samples that have been tested accordingly to ISO 12312-1.

**Methods:**

The procedure is in accordance with the resistance to ignition test. It consists of heating a steel rod to 650 °C and pressing it against the sample surface for 5 s, with a force equivalent to the rod weight. For carrying out the assessments, we have build resistance to ignition testing system and assured the testing requirements of the standard. The apparatus has an electrical furnace with a temperature acquisition circuit and electronic control that maintains the temperature of the steel rod at 650 °C. A linear actuator was designed for the project to drive the steel rod vertically and pressing it against the sunglasses samples. The control system is composed by a Freescale development board FRDM-KL25Z with an ARM Cortex-M0 embedded. We have also provided a LabView PC interface for acquiring, displaying, and storing data as well as added a physical control panel to the equipment for performing the evaluations. We assessed 410 sunglasses frames at the built apparatus, where the 410 lenses came out to be in accordance with the guidelines provided by the ignition to resistance test. Out of the 410 tested frames, 50% were made of polyamide (nylon 12); 10% were made of polyamide (nylon 11, mamona oil); 5% were made of cellulose acetate; 15% were made of ABS and 20% were made of polycarbonate. Out of the 410 tested lenses, 80% were polycarbonate; 2% were polymethyl methacrylate (PMMA); 5% CR-39 (with polarizing filter inside); 12.8% polyamide; 0.2% glass.

**Results:**

For all the 410 tested spectacles frames and lenses, none burst into flames or continued to melt at the end of the procedure, being in compliance with ISO 12312-1:2013.

**Conclusions:**

The evidences show that all the tested thermoplastic and thermosetting materials are exceptionally resistant to ignition and all samples assessed comply with the resistance to ignition test. The analysis of the sunglasses made herein assures that most of sunglasses currently available to population are made of safe material.

## Background

Cellulose nitrate, during the 50 and 60s, was the most used material in the manufacturing of glasses. It was ideal for the frames because it presents high hardness and durability, besides being able to maintain its shape and color for long periods. Due to safety issue, being highly flammable, its use was discontinued in the 1980s [1]. The successor of cellulose nitrate was cellulose acetate, which although less flammable, lost its market in the 90 s after the adoption of a heated rod for the resistance to ignition test. Although the flame propagation in this material was slow, the risk to the wearer was in the ignition of clothing and hair, and could be fatal.

In the 70s, glass was heavily used in sunglasses lenses. An Australian study published in this period evaluated the ignition resistance of the main materials used in the manufacture of glasses. Cellulose nitrate has been classified as extremely flammable. Cellulose acetate, cellulose acetate butyrate and nylon (polyamide) were categorized as resistant to flame propagation. The materials that were the most resistant to ignition were polycarbonate and PVC, considered self-extinguishable [2, 3].

There are records of major fires in eyeglass frame it is believed that all were directly related to the use of cellulose nitrate due to the common characteristics of the fires: they started unexpectedly and the flames spread rapidly [1].

Currently the typical materials that sunglasses are made belongs to two distinct categories: thermoset and thermoplastic. Thermoplastics can be melted back into a liquid, whereas thermoset plastics always remain in a permanent solid state. Thermoset plastics contain polymers that cross-link together during the curing process to form an irreversible chemical bond [4].

Thermoset used for frames is nitrocellulose and for lenses is allyl diglycol carbonate (CR-39). Thermoplastic used for frames are polyamides (or nylons), polycarbonates, cellulose acetate, cellulose propionate acetate, cellulose acetate butyrate and acrylonitrile butadiene styrene (ABS). For lenses, polycarbonate and polyamide copolymers. The ignition temperature for these typical materials are listed on Table 1. The most widely used for polarized lens glasses is polarising film cast into CR-39.

**Table 1:** Melting point and self ignition temperatures for typical material of sunglasses frames and lenses

Material	Melting point temperature (°C)	Self-ignition temperature (°C)
Polyamides (or nylons)	172–260	424–532 [5]
Polycarbonates	140–150 [8]	580 [8]
Cellulose acetate	49–121 [8]	475–540 [6, 8]
Cellulose propionate acetate	188–210 [7]	432 [7]
Cellulose acetate butyrate	127–240 [7]	–
ABS—acrylonitrile butadiene styrene	88–25 [8]	416 [8]
Cellulose nitrate (or nitrocellulose)	160 [9]	141 [6]
CR-39 (allyl diglycol carbonate)	129 [10]	443 [10]
PMMA—polymethyl methacrylate (acrylic)	91–125 [8]	452–460 [6]

Thus, with the history of flammability in sunglasses, the standards provide a test that ensures that certified glasses are not flammable when exposed to the sources of heat that wearers are subjected in everyday life.

Sunglasses’ standards urge to provide adequate protection to users of spectacles against injuries and secondary hazards caused by sunglasses wearing. Under the given conditions, sunglasses standard provides protection in order to avoid undesirable secondary risks [11].

This work is part of a continuous research being conducted in the group, in Brazil [12–14], in order to contribute to standards’ requirements and ISOs regarding sunglasses, since the tests and requirements have been established decades ago. In addition, the profiles of users and fashion styles, as well as the material which sunglasses are made of, are continuously changing.

Australia was the first country to issue a standard for sunglasses for general use in 1971, the AS 1067. Until this release, only occupational or industrial spectacles use were regulated [11]. Currently, the latest version was published in 2006 and it is a joint between Australia and New Zealand standards, the AS/NZS 1067, named “sunglasses and fashion spectacles” [15].

The United States was the second, in 1972, with the ANSI Z80.3. The final version dated 2015: “nonprescription sunglasses and fashion eyewear requirements” [16].

Only in 1997 the official European standard for sunglass, the EN 1836 [17], was adopted. Its latest revision was published in 2013 and EN 1836 has been replaced by the following two standards: EN ISO 12311: 2013—personal protective equipment—test methods for sunglasses and related equipment and EN ISO 12312 -1: 2013—eye and face protection—sunglasses and related eyewear—part 1 sunglasses for general use.

The standard recommended optical and non-optical characteristics to sunglasses, also describing procedures to evaluate performance. This piece cited the flammability test procedure and this method is fully described in the 2002 released standard EN 168—“personal eye-protection—non-optical test methods”. Recently, the two new ISO standards published in 2013, refer to sunglasses: ISO 12311, “personal protective equipment—test methods for sunglasses” [18]; and ISO 12312-1 [19], designated “eye and face protection—sunglasses and related eyewear—part 1: sunglasses for general use”.

The first sunglass Brazilian standard ABNT NBR 15111 was released in 2003 [20]. It was a mirror from European standard EN 1836. In 2013, Brazilian committee released a new version of ABNT NBR 15111, where author Liliane Ventura was a member and the major revision established the 400 nm as upper limit for ultraviolet protection. However, in 2015, Brazilian standard was revoked and 380 nm upper limit for ultraviolet was set back again, since it became a mirror of ISO 12312-1, now designated ABNT NBR ISO12312-1 [21]. Despite all these changes, one of the requirements remained unchanged: the resistance to ignition test for sunglasses, which this work is focused. Resistance to Ignition testing is quite important, since sunglasses should be protected from burning into flames, if cigarette sparks reach the spectacles, as ISO 12311:2013 and ISO 12312-1:2013 (Brazilian standard NBR 12312-1:2015 mirror standard) recommend sunglasses manufacturers to protect consumers from sun glare, harmful radiation, and secondary hazards as flammability. The resistance to Ignition test procedure, also known as resistance to ignition test, is entirely described in the NBR ISO 12312-1:2015 and was completely based on the European standard EN 168. Since there are no mandatory assessments for sunglasses in Brazil, evaluating sunglasses’ performance to flammability envisions the scenario of sunglasses worn in Brazil into compliance with the ignition test’s directive. This paper describes the built apparatus to perform resistance to ignition tests and the results for 410 sunglasses.

## Methods

In order to be able to run the resistance to ignition test, we have developed and built an apparatus for testing the samples, which complies with ISO 12312-1:2013 and ISO 12311:2013 recommendations.

The apparatus heats the end of a steel rod to the temperature of 650 ± 20 °C over a length of at least 50 mm. A thermocouple is attached at a distance of 20 mm from the hot face of the steel rod to assess the temperature. Subsequently, the heated face of the steel rod is pressed against the surface of the sunglass sample for 5 s with a force equivalent to the rod weight and then it is removed. All tests must be performed in an environment with a temperature of 23 °C. To verify whether the sample ignites or continues to glow, visual inspection is carry out during the procedure.

### Procedure

All tests procedures comply with directives ISO 12312-1:2013 and ISO 12311:2013 recommendations for the flammability test and are described as follows:

First, the equipment heats the end of a steel rod to the temperature of 650 ± 20 °C over a length of at least 50 mm. A thermocouple should be attached at a distance of 20 mm from the hot face of the steel rod to assess the temperature. Next, the machine presses the heated face of the steel rod against the surface of the sunglass sample for 5 s with a force equivalent to the rod weight and then removes it. All tests must be performed in an environment with a temperature of 23 °C. To verify whether the sample ignites or continues to glow, visual inspection is carry out during the procedure.

### Apparatus

**Fig. 1:**
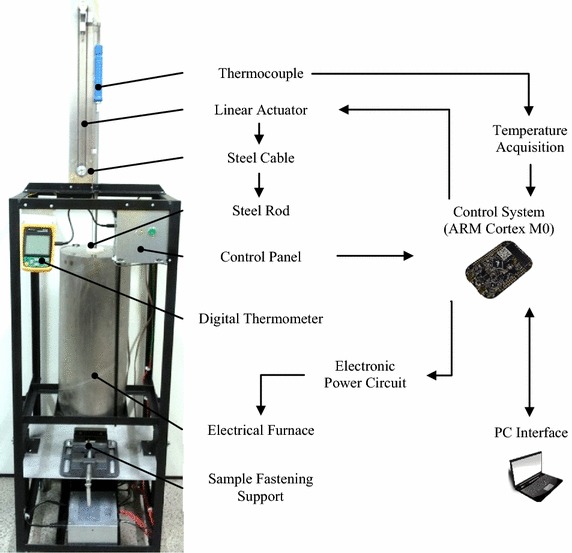
Apparatus schematic diagram for performing the resistance to ignition tests in sunglasses

Figure 1 shows the schematic diagram of the built apparatus. In summary, the apparatus is composed by an electrical furnace that maintains the temperature of the steel rod at 650 ± 20 °C; a linear actuator that drives the rod vertically against the sunglass sample surface for 5 s; and a control panel with a PC interface to acquire and store data.

### Heat source and temperature control

The electrical furnace can reach temperatures up to 1000 °C and it is made of ceramic with an insulation of rock wool covered by an aluminium housing. The electrical resistance, with a length of 6 m and diameter of 2.2 mm, is an alloy composed of iron, chromium, and aluminium in which the resistance value varies < 4% at 1000 °C.

The temperature feedback is given by a K-type thermocouple attached inside the furnace at a distance of 20 mm from the hot end of the steel rod. A thermometer with temperature display assesses the temperature measured by the thermocouple and sends it via serial RS-232 to the control system.

**Fig. 2:**
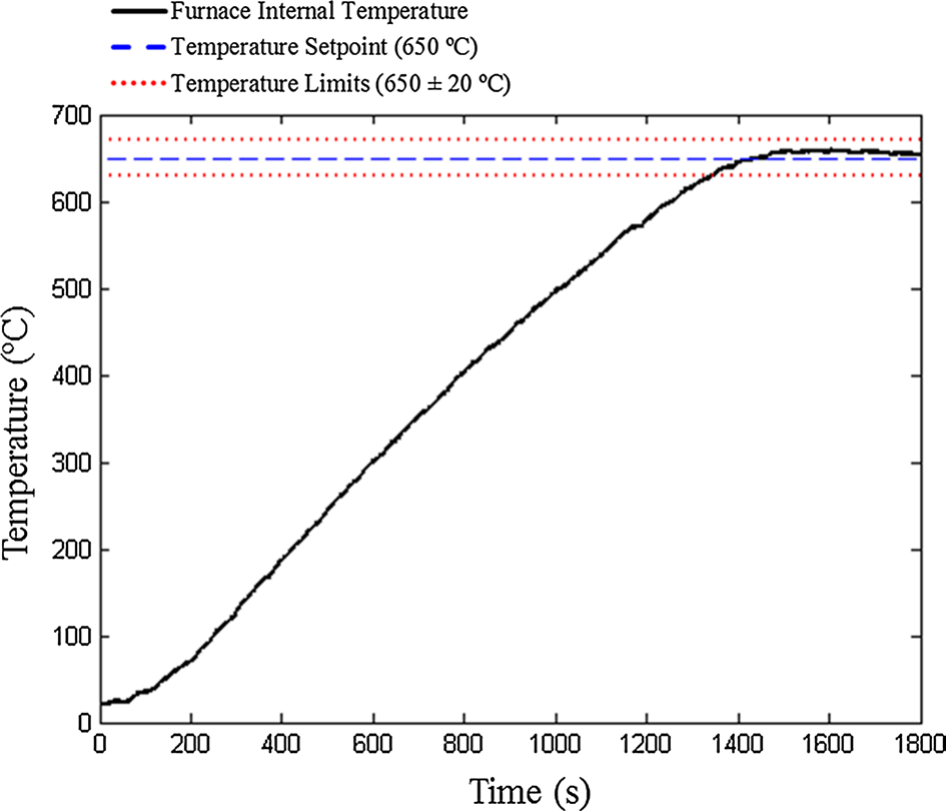
Furnace internal temperature response

To set the temperature in 650 ± 20 °C, a PID controller commands the power dissipated by the resistance inside the furnace from zero to 1697 W. A TRIAC BTA41 was used in ON–OFF mode to drive power to the electrical resistance. The standard NBR ISO 12312-1:2015 recommends a maximum temperature assessment error of ± 10 °C and a working temperature for the hot steel rod within the range of 630 and 670 °C. For the equipment developed, the maximum temperature assessment error is ± 3.95 °C at 650 °C, which is smaller than the maximum recommended and thus satisfy this requirement. Figure 2 exhibits the furnace internal temperature response. The apparatus achieves the working temperature in 1320 s (22 min) and then the resistance to ignition tests can be performed. Moreover, the PID controller maintains the temperature within the boundaries of 630 and 670 °C during its operation.

For protecting and insulating the low-voltage circuits, such as the control system, we applied a MOC3041 opto coupler.

### Steel rod and linear actuator

The steel rod is made of stainless steel AISI 304, has nominal dimensions of 6 mm diameter and 300 mm length, with flat end faces and perpendicular to its longitudinal axis.

For moving the steel rod vertically during the tests, we built a linear actuator connected to the rod by a steel cable. The actuator is composed by an IC DRV8825 that drives the hybrid stepper motor of 6.0 V and 1.2 A per phase. We coupled a timing pulley to the stepper shaft to run a timing belt tensioned by a spring-loaded pulley.

### Control system and interface

The control system consists of a freescale development board FRDM-KL25Z with an ARM Cortex M0 microcontroller. It controls the Resistance to Ignition test system and it is connected to a PC interface via USB connection.

We have made user interface in LabView to control the equipment from the computer, for acquiring, displaying, and storing the data. In addition, a physical control panel was developed and placed in the equipment for performing the resistance to ignition tests.

### Testing the samples

**Fig. 3:**
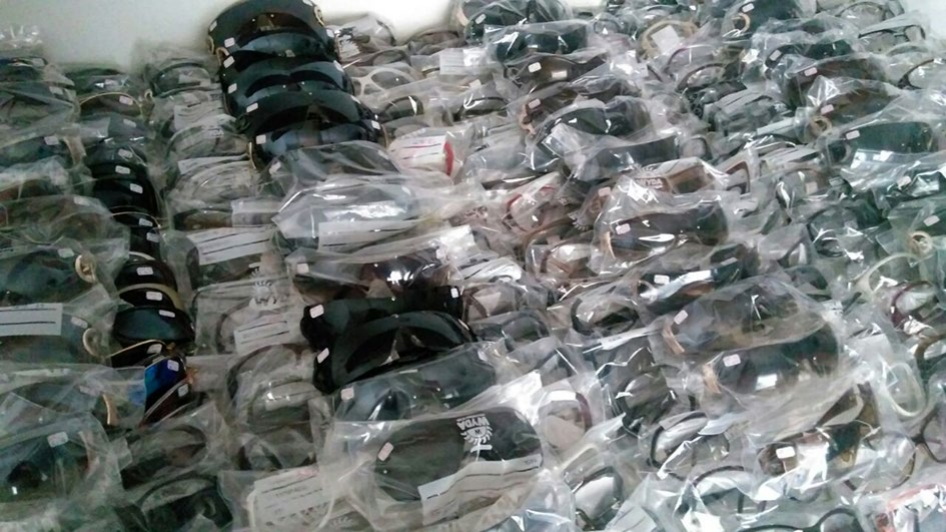
Sunglasses samples. Some of the lenses were already removed for tests

We have received as donation from an association of manufacturers of sunglasses the majority of the tested samples. Figure 3 shows the samples. We have labelled them and after the resistance to ignition tests we have determined the material of lenses and frames with help of chemical engineers and chemists, by the traditional methods [4] by scratching the material, setting fire and observing the flame (its colour and if it was extinguished or continued to burn) and smoke (its colour and smell).

Out of the 410 tested frames, 50% were made of polyamide (nylon 12); 10% were made of polyamide (nylon 11, mamona oil); 5% were made of cellulose acetate; 15% were made of ABS and 20% were made of polycarbonate. Out of the 410 tested lenses, 80% were polycarbonate; 2% were polymethyl methacrylate (PMMA); 5% CR-39—allyl diglycol carbonate—(with polarizing filter cast inside); 12.8% polyamide; 0.2% glass.

In a set of 410 lenses tested, 409 were made of copolymers (polycarbonate and polyamide) and CR-39, while one was made of glass.

## Results and discussion

One of the challenges of this work was developing the apparatus for testing the samples, which gives us complete control of all information needed for research purposes. There is no certification lab in Brazil for sunglasses and the intention of this work, besides studying the compliance of commonly types of sunglasses being sold in this country, with respect to the resistance to ignition test, is to provide means for testing these samples, by building such an apparatus.

The developed apparatus is in accordance with all requirements of current Brazilian standard NBR ISO 12312-1:2015. The apparatus reaches the temperature of 650 ± 20 °C in 22 min of operation and then can perform the ignition procedures continuously, as shown in Fig. 2.

Further, we evaluated 410 spectacles with the flammability test: all sunglasses samples had a lens and a frame assessed using the equipment development. All tests were carried out according to ISO 12311:2013 recommendations.

### Apparatus

The built equipment complies with all ISO 12311:2013 recommendations for the Resistance to Ignition test. It has a maximum temperature assessment error of ± 3.95 °C at 650 °C, smaller than the maximum suggested of ± 10 °C and maintains its working temperature within the range of 630 and 670 °C during the procedures. A length of at least 50 mm of the steel rod should be heated and the apparatus heats the completely the steel rod for performing the tests. In addition, the thermocouple is attached at 20 mm from the hot face of the steel rod for assessing the temperature. The environment temperature is maintained at 23 °C and we carry out visual inspection during the procedures to verify whether the samples burst into flames or continue to glow after the rod removal.

### Testing the frames of sunglasses

**Fig. 4:**
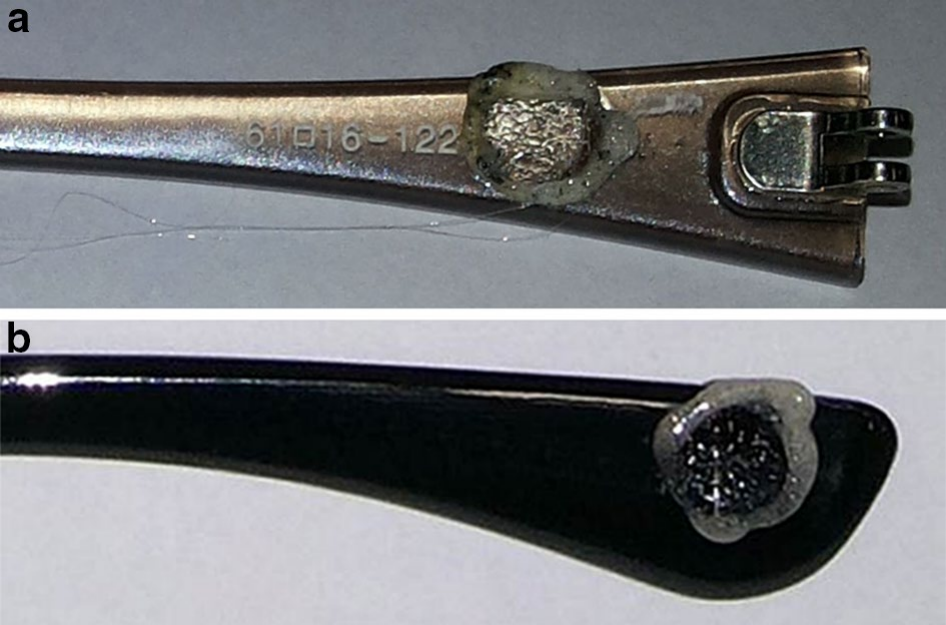
Sunglasses frames subsequent to the resistance to ignition test

The outcome for the 410 polycarbonate frames was similar. The heated steel rod used during the tests partially melted the frames in the contact region, as illustrated in Fig. 4 for distinct samples. Any of the pieces burst into flames or continued to melt at the end of the procedure.

### Testing the lenses of sunglasses

**Fig. 5:**
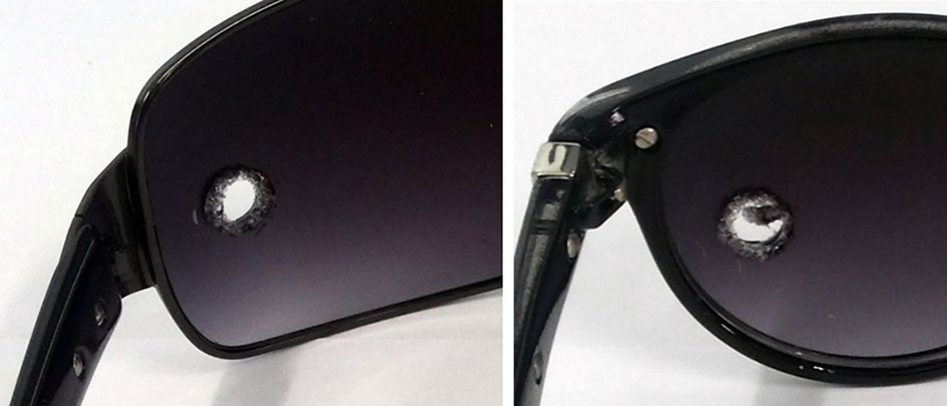
Sunglasses lenses subsequent to the resistance to ignition test

For 409 lenses (except the one made of glass), the heated steel rod melted the polymer in the region of contact with all samples. Figure 5 shows two lenses after the resistance to ignition test. Only the glass lens remained undamaged after the procedure. Moreover, none sample ignited nor kept melting after the hot rod removal.

Based on the results we could conclude that all sunglasses samples evaluated are in accordance with the standard ISO 12311:2013, therefore with standard NBR ISO 12312-1:2015 and are not flammable. As expected, the glass has a melting temperature greater than 1200 °C and should not be damaged by the rod at 650 °C. Contrary, other materials do melt, as expected by Table 1 data.

Furthermore, the results presented here are aligned with the outcomes of an Australian study that investigated flammability in common materials used in industrial and cosmetic spectacles.

## Conclusions

In conclusion, the developed equipment is in accordance with all recommendations made by the standard NBR ISO 12312-1:2015 for performing the resistance to ignition test procedure. The apparatus was efficient for carrying out the tests and was essential for this work.

From the 410 lenses and 410 frames assessed, none sample ignited or continued to melt after the removal of the rod at 650 °C.

All tested samples here may represent the types of sunglasses sold in Brazil. All of them comply with ISO and Australian resistance to ignition standards, and in addition, it is flame-resistant and self-extinguishing [2].

The analysis of the sunglasses made herein assures that most of sunglasses currently available to population are assured to be made of safe material.
